# A New Paradigm in Managing Advanced Ovarian Cancer: Differentiating Patients Requiring Neoadjuvant Treatment from Primary Cytoreduction

**DOI:** 10.3390/cancers13194925

**Published:** 2021-09-30

**Authors:** Francois Kraus, Houssein El Hajj, Marie-Cécile Le Deley, Othman Aissaoui, Bertrand Gachon, Annick Chevalier, Cyril Abdeddaim, Anne-Sophie Lemaire, Mariem Ben Haj Amor, Dienabou Sylla, Eric Leblanc, Fabrice Narducci, Delphine Hudry

**Affiliations:** 1 Department of Obstetrics and Gynecology, University Hospital Center, 80000 Amiens-Picardie, France; kraus.francois@chu-amiens.fr; 2 Department of Gynecologic Oncology, Oscar Lambret Center, 59000 Lille, France; o-aissaoui@o-lambret.fr (O.A.); bertrand.gachon@univ-poitiers.fr (B.G.); a-chevalier@o-lambret.fr (A.C.); c-Abdeddaim@o-lambret.fr (C.A.); e-leblanc@o-lambret.fr (E.L.); f-narducci@o-lambret.fr (F.N.); d-hudry@o-lambret.fr (D.H.); 3 Clinical Research and Innovation Department, Oscar Lambret Center, 59000 Lille, France; m-ledeley@o-lambret.fr (M.-C.L.D.); s-marchant@o-lambret.fr (D.S.); 4 Centre de Recherche en Epidémiologie et Santé des Populations, INSERM, Paris-Sud, Paris-Saclay University, 94800 Villejuif, France; 5 Department of Pathological Anatomy and Cytology, Oscar Lambret Center, 59000 Lille, France; a-lemaire@o-lambret.fr; 6 Department of Radiology, Oscar Lambret Center, 59000 Lille, France; m-BenHajAmor@o-lambret.fr; 7 Laboratoire de Protéomique, Lille University, Inserm, U-1192-Protéomique Réponse Inflammatoire Spectrométrie de Masse-PRISM, 59000 Lille, France

**Keywords:** advanced ovarian cancer, primary cytoreduction, neoadjuvant chemotherapy, interval debulking surgery, propensity score

## Abstract

**Simple Summary:**

This study evaluates the comparability of patients treated with primary cytoreduction and patients undergoing neoadjuvant chemotherapy for advanced stages high grade serous ovarian carcinoma by comparing the preoperative and postoperative characteristics after a propensity score matching analysis during ten years in a tertiary cancer center.

**Abstract:**

Our study aims to evaluate the comparability of primary debulking surgery (PDS) and neoadjuvant chemotherapy (NACT) patients. This single-center retrospective study includes all patients treated for advanced stages high-grade serous ovarian carcinomas (HGSOC) between 2007 and 2017. Preoperative characteristics and postoperative outcomes were compared after a propensity score matching analysis. Of the 221 patients included, 38% underwent PDS, and 62% received NACT. There was no age difference at diagnosis; however, CA125 levels, PCI score levels, and rates of stage IV were higher in the NACT group. There were no differences concerning the rate and the severity of complications (*p* = 0.29). The propensity score distribution showed a broad distinction between PDS patients and NACT patients with no significant overlap. Survival analyses demonstrate, after a median follow-up of 66.5 months, an overall survival (OS) of 105.9 and progression-free survival (PFS) of 29.2 months in the PDS group, compared to OS of 52.8 and PFS of 18.9 months in the NACT group. Advanced HGSOC is a heterogeneous population, in which inoperable patients should be differentiated from PDS patients based on many factors, primarily tumor burden.

## 1. Introduction

Ovarian carcinoma represents the fifth cause of cancer-related mortality in women. Due to its pauci-symptomatology, patients tend to present at advanced disease stages (IIIC and IV), which contributes to the reduced 5 year overall survival (OS) rate (46%) [1,2,3,4]. The incidence of ovarian cancer remained stable over the last 30 years [5,6]. This was associated with improved 5 year survival for all epithelial ovarian cancer (EOC) stages (42% and 26% for FIGO stages III and IV, respectively) but no improvement in the 10 year OS (24% all stages combined). This reflects a better disease control but no improvement in long-term survival [7,8]. Low long-term survivorship is, in part, related to the disease burden at diagnosis and the emergence of chemoresistant clones that contribute to disease recurrence in 70 to 90% of the cases [9]. Almost 90% of ovarian cancers are EOC, of which HGSOC constitutes the vast majority and accounts for nearly 70% of deaths [10,11]. Treatment modalities for advanced high-grade serous ovarian carcinomas (HGSOC) remain a serious subject of debate. They include either complete PDS or interval debulking surgery (IDS) associated with carboplatin and paclitaxel-based chemotherapy with or without bevacizumab and or targeted treatment is the standard of care for EOC [12,13].

Studies have shown that optimal cytoreduction (CC) is associated with enhanced survival rates [14]. The European Society of Gynecologic Oncology highlighted the importance of PDS with CC in 2016 and 2020 by requiring a PDS rate greater than 50% for stage III–IV EOC as a quality indicator for ovarian cancer centers [15]. CC is sometimes associated with increased morbidity and is undesirable when this morbidity outweighs the benefits of surgery [16]. Therefore, retrospective and prospective trials analyzed outcomes of PDS and IDS. Recent randomized controlled trials (RCTs) and a Cochrane review concluded the non-inferiority of NACT and IDS compared to PDS. However, RCT faced prominent critics concerning the aggressiveness, rate of CC, and decreased survival in the CHORUS, EORTC, and JCOG0602 trials [17,18,19]. The SCORPION trial addressed these critics, notably the low rate of CC, but presented low rates of patient accrual. It concluded no difference in progression-free survival (PFS), with a significant decrease in postoperative morbidity in the NACT group [20].

It is, however, important to highlight that RCTs and observational studies are not comparable, but rather complementary. RCTs present an important internal and limited external validity, while observational studies show a better external validity with limited internal validity [21]. When comparing RCTs and observational studies in ovarian cancer management, we notice that patients included in RCTs comparing PDS and IDS were suitable for both treatment plans. In contrast, in real-life practice, and according to the guidelines, only patients who cannot undergo PCS will receive NACT reflecting a poorer patient status in the NACT group.

However, the objective of achieving high PDS rates does not consider the intrinsic disease-related characteristics nor the specificity of each patient’s condition and disease burden.

The main objective of this study is to compare the real-life populations of advanced stages EOC patients (PDS and NACT populations) treated over ten years in a high-volume tertiary cancer center. Subsequently, we evaluate whether a new patient-centered approach could be adopted in treatment modality attribution.

## 2. Materials and Methods

This is a retrospective single-center study at a tertiary cancer center performed between 2007 and 2017. The study protocol was approved by the institutional review board and conformed to the French ethical standards and 2008 Helsinki declaration.

All patients presenting confirmed newly diagnosed FIGO 2018 stages IIIC or IV HGSOC were included in the study. Patients underwent a complete workup, comprising imaging (thoraco-abdominopelvic CT scan), biologic workup (including CA125), and diagnostic laparoscopy, to evaluate the disease burden and resectability (using the Peritoneal Carcinomatosis Index (PCI)). All patients underwent biopsies that expert pathologists analyzed.

Assigning patients to either PDS or NACT was based on several criteria that remained unchanged during the study period. Contraindications for surgery were either patient or disease related.

Patient-related contraindications include altered general status (low-performance status (PS)), comorbidities, severe malnutrition. Advanced age was also taken into consideration.

Disease-related contraindications were evaluated by imaging (disease localization), laparoscopy, or both, and included deep infiltration of the small bowel mesentery, diffuse carcinomatosis requiring more than two bowel resections or leading to short bowel syndrome, stomach, duodenal, or pancreatic resections, multiple liver or lung metastasis, suprarenal trunks involvement (coeliac trunk, hepatic trunk, and left gastric artery), brain metastasis, and unresectable diffuse lymph node metastasis.

To limit the bias in triaging patients to NACT or PDS, the multidisciplinary tumor board performed a systematic and thorough evaluation of the patient’s file following the ESGO 2017 recommendations before validating the decision. This includes evaluating the patient’s related contraindications and disease burden (reviewing imaging, operative reports, and photos taken during diagnostic laparoscopy) [22,23]. All surgeries were performed by experienced surgeons with the intention of achieving complete cytoreduction. Based on their baseline characteristics (disease burden and personal health status), all patients were attributed to either PDS or NACT. During the treatment course of the patients attributed to NACT, four subcategories appeared based on the disease response to NACT, dictating the timing of surgical cytoreduction. Patients not eligible for PDS due to disease extent, severe comorbidities, or low-performance status (PS), underwent paclitaxel-carboplatin-based NACT. Patients presenting stage IV disease underwent a systematic clinical, biological, and radiological evaluation of the treatment efficacy at three cycles. Most of the stage IV patients underwent surgery after 6 NACT cycles to ensure the treatment of extra-abdominal metastasis. However, stage III patients underwent NACT with an evaluation every three NACT cycles (CA125 levels, CT scan, and diagnostic laparoscopy). This is followed by cytoreduction, unless imaging or laparoscopy showed contraindications. Some patients continued to progress or to alter their status despite NACT and were never eligible for IDS.

Clinical and pathological data were collected based on a predefined data dictionary. The following data were collected: age at diagnosis, body mass index (BMI), PS, disease characteristics at diagnosis (CA125, PCI, histological type, grade, cytology, ascites, and FIGO stage), characteristics of surgery (date, procedures performed, strategy (PDS, IDS), completeness of the cytoreduction (CC score), and Aletti’s surgical complexity score), and characteristics of the systemic treatment (neoadjuvant or adjuvant chemotherapy, number of cycles, protocols) [24,25].

Patients were stratified into two categories based on the treatment strategy defined based on the initial patients and disease characteristics during the tumor boards: PDS and NACT groups. The NACT group was further divided into three subgroups based on the number of cycles required before surgery was possible: early surgery <6 NACT cycles, surgery at 6 NACT cycles, and delayed surgery >6 NACT cycles. Surgeries were classified into standard, radical, and supra-radical according to the Pomel classification, in which the standard surgery includes hysterectomy, bilateral salpingo-oophorectomy, pelvic peritonectomy, omentectomy, appendectomy, pelvic and/or para-aortic lymphadenectomy; radical surgery includes the addition of recto-sigmoid resection, and supra radical surgery includes diaphragmatic peritonectomy, liver resection, splenectomy, cholecystectomy, and other digestive tract resections [26]. Complete cytoreduction (CC0) was defined as the absence of any macroscopic residual disease at the end of surgery. Postoperative residual disease was stratified according to the remaining disease after surgery and was CC1 <0.25 cm, CC2 <2.5 cm, and CC3 >2.5 cm [24].

Surgery-related complications were evaluated during the hospital stay, at one and two months postoperatively. Follow-up visits were planned for one month after surgery, then every four months for five years. During follow-up visits, patients underwent gynecologic examination, CA125 level, and, if needed, thoraco-abdominopelvic CT scan. Complications were stratified according to the Clavien–Dindo classification [27,28].

OS and PFS were defined as the duration from initial laparoscopy to death from any cause and from the initial laparoscopy to any recurrence or progression of cancer or death from any cause, respectively. The local tumor board confirmed disease progression and relapse based on clinical, biological, and radiological assessment. Data were censored for patients alive at the end of the study without progression or relapse.

We performed a descriptive analysis of the whole cohort, PDS, and NACT groups, respectively. Quantitative variables were represented by median, minimum, and maximum values. Numbers and percentages represented categorical variables. Characteristics of patients in the PDS and NACT groups were compared using student’s t-test for quantitative and chi-square of Fisher’s exact test for categorical variables.

While estimating the association between survival and treatment modality (NACT versus PDS) using Cox models, an indication bias might occur as treatment choice might depend on initial characteristics. To limit this bias, Cox models were stratified on the quintiles of the propensity score (the conditional probability to receive a treatment given the initial characteristics). This propensity score is obtained by logistic regression of treatment modality according to initial characteristics (an ordinal logistic regression was used when analyzing treatment in three groups of surgery timing: <6 cycles of NACT, 6 cycles, >6 cycles). Stratification on propensity score quintiles requires that patients from each treatment group be represented in each quintile. In the case of non-overlap of treatment groups across all the quintiles of the propensity score, patients from the quintile with no representation of all treatment groups were excluded from the analysis [29]. This model was also performed to analyze the survival data when comparing the PDS and the early NACT groups (<6 cycles). This early NACT group represents the grey zone between PDS and NACT and causes the most significant doubt on whether patients should undergo surgery or NACT. A third similar analysis was performed to compare the different NACT subgroups.

All statistical tests were two-sided with a 5% level of significance and 95% confidence intervals. The analyses were performed using Stata/SE (version 13.1) statistical software (StataCorp LP, College Station, TX, USA).

## 3. Results

The initial cohort included 254 patients, of which 221 met the inclusion criteria. Table 1 shows patients’ and disease characteristics. The median age at diagnosis was 63.2 years, with no difference between the groups. The rate of patients with a PS = 1 was twice higher in the NACT group (20.9% versus 10.7%) with a *p* = 0.08. Mean CA125 levels were significantly higher in the NACT group, valued almost twice as elevated than the PDS group (2249 versus 1077) *p* = 0.002. Additionally, 181 (81.9%) and 40 patients (18.1%) had FIGO stage IIIC and IV disease, respectively. Compared to the PDS group, patients in the NACT group presented a statistically significant higher rate of FIGO stages IVA (13.9% versus 2.4%) and IVB (11.6% versus 3.6%), *p* = 0.001. The median PCI for the entire population was 20 (0–39). Mean PCI scores were almost twice higher in the NACT group (24.4 versus 10.6), *p* = 0.0001. No difference was found in the ascites rates (*p* = 0.19).

The CA125 level, FIGO stage, and PCI distributions show that NACT patients presented significantly poorer risk factors. However, no significant difference was found for the age at diagnosis and BMI.

The anatomopathological diagnosis was performed by laparoscopy for most patients (*n* = 82, 97.6% in the PDS group, and *n* = 120, 87.6% in the NACT group). A diagnosis via radiological biopsy was more frequently performed in the NACT group (12.4%, *n* = 17, versus 2.4%, *n* = 2).

Of the 221 patients, 84 (38%) underwent PDS, and 137 (62%) required NACT. Additionally, 94% (n = 129) of the NACT patients underwent IDS, and 8 patients were never eligible for cytoreduction. In the IDS group, 50 patients, 50 patients, and 29 patients underwent surgery early surgery (<6 NACT cycles), surgery at 6 NACT cycles, and delayed surgery (>6 NACT cycles), respectively.

As shown in Table 2, no significant difference was found in the PCI scores at the time of surgery, with a median PCI of 10 for both groups, *p* = 0.34. The PDS group presented a rate of radical surgery twice higher than the rate in the NACT group (26.2% versus 10.9%), *p* = 0.01. Furthermore, the PDS group presented a higher rate of bowel resection (56% versus 34.9% in the NACT group), *p* = 0.002. There were no significant differences in Aletti’s score and the rate of para-aortic lymphadenectomy *p* = 0.82 and *p* = 0.62, respectively. A low Aletti’s score was found in 5 patients (2.3%), while intermediate and high complexity scores were found in 117 (54.9%) and 91 (42.7%), respectively.

CC0 was achieved in 193 (90.6%). One patient (0.5%) was CC3 postoperatively. A higher rate of surgical completeness was achieved in the PDS group (*p* = 0.001). No significant differences were found concerning the duration of surgery (*p* = 0.48), blood loss (*p* = 0.75), transfusion rate (*p* = 0.1), and duration of hospital stay (*p* = 0.58), with a mean stay of 5.5 days and 6.3 days in the PDS and NACT groups, respectively.

No treatment-related deaths were recorded. Only 20 patients (9.4%) presented intraoperative complications with no difference between the two groups (*p* = 0.96). The most frequent intraoperative complication was hemorrhage in 5.2%, followed by bowel injury, which was 1.9% requiring bowel resection. Diaphragmatic injury during peritonectomy, bladder injury, and anesthesia-related complications occurred in three, one, and one patients, respectively.

Sixty-one patients (28.6%) presented postoperative complications, of which 15.5% were mild (grade 1–2), requiring no or only pharmacological treatment. The most frequent complications were infection (*n* = 26), lymphocele (*n* = 16), bowel obstruction (*n* = 6), fistula (*n* = 5), and hemorrhage (*n* = 4). No significant difference was found concerning the rate and severity of perioperative complications (*p* = 0.29).

**Figure 1:**
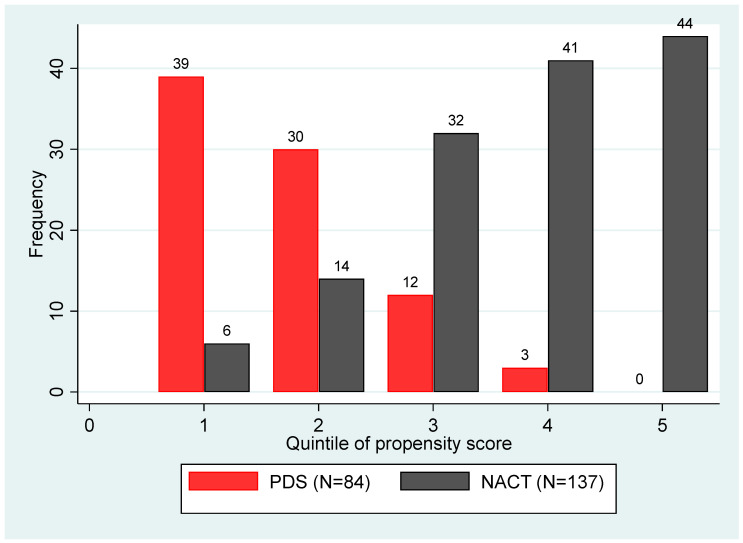
A propensity score for PDS versus NACT.

A propensity score matching was used to reduce the bias when evaluating the association between survival and treatment groups (NACT versus PDS groups). Variables used in the matching included age, FIGO Stages, presence of ascites, and PCI score at diagnosis. Baseline CA125 level was not included owing to the significant correlation to baseline PCI (*p* = 0.02) and missing data in 13 patients. The propensity score distribution showed a broad difference between patients treated with PDS and patients treated with NACT with no significant overlap. Figure 1 shows that all patients with the highest score (*n* = 44) belong to the NACT group.

The propensity score included age at diagnosis, FIGO stage, ascites at diagnosis, and PCI at diagnosis. The baseline CA125 level was not included in the propensity score matching due to the significant correlation to the baseline PCI index (*p* = 0.02) and the missing data in 13 patients. For patients with missing data for PCI at diagnosis (all in the NACT group), we imputed the mean value of the PCI in the NACT group (mean = 24.4). The equation of propensity score is:

$$\begin{array}{c} Propensity\;score=P\left(NACT|age10,FIGO.stage,ascites,\;PCI10\right)=\frac{\exp\left(X\right)}{1+\exp\left(X\right)}\;with\\ \begin{array}{cc} X= & -3.249-0.108451\times age10+3.325978\times FIGO.stage.4A\\ \; & +2.55267\times FIGO.stage.4B-0.9026239\times ascites+2.502814\times PCI10 \end{array} \end{array}$$

where:

age10 = age at diagnosis divided by 10;
  FIGO.Stage.4A = 1 if FIGO stage = 4A, FIGO.Stage.4A = 0 in other cases;
  FIGO.Stage.4B = 1 if FIGO stage = 4B, FIGO.Stage.4B = 0 in other cases;
  Ascites = 1 if there are ascites at diagnosis, else Ascites = 0;
  and PCI10 = Sugarbaker Peritoneal carcinosis index at diagnosis, divided by 10.

Since the propensity score quintiles and the treatment groups (PDS and NACT) did not entirely overlap, we restricted the analysis by excluding the highest quintile. In a Cox model estimated using the resulting selected subset and stratified by the quintile of the propensity score, the HR of progression associated with NACT versus PDS was 1.62 (95%CI, 1.01–2.61; *p* = 0.046), and the HR of death was 2.45 (95%CI, 1.27–4.73; *p* = 0.008).

**Figure 2:**
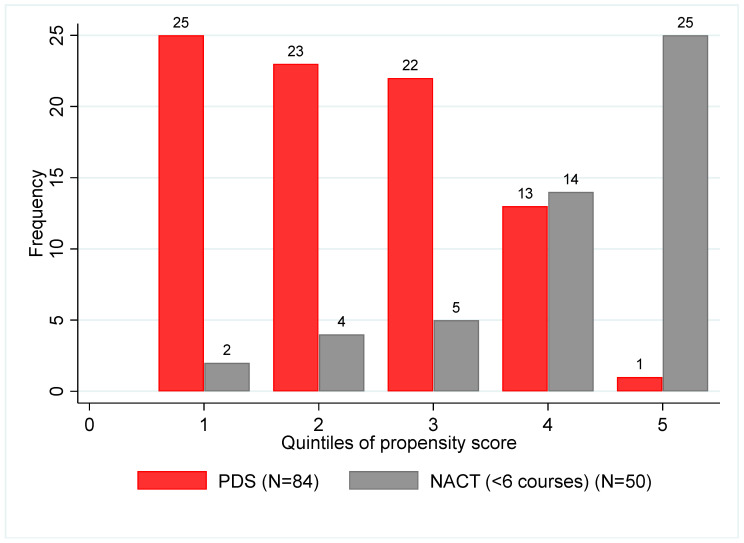
A propensity score for PDS versus early IDS surgery (<6 NACT cycles).

A propensity score matching was used to reduce the bias when evaluating the association between survival and treatment groups when comparing the PDS group and the early IDS surgery (<6 NACT cycles). Variables used in the matching included age, FIGO Stages, presence of ascites, and PCI score at diagnosis. Since both treatment groups are represented in each quintile of the propensity score, we were able to perform a Cox model stratified on the quintiles of the propensity score without having to exclude patients (Figure 2).

The equation of propensity score is:

$$\begin{array}{ll} Propensity\;score & =P\left(“NACT:<6\;courses”|age10,FIGO.stage,ascites,\;PCI10\right)\\ & =\frac{\exp\left(X\right)}{1+\exp\left(X\right)} \end{array}$$

with

$$\begin{array}{cc} X= & -4.250162-0.0089731\times age10+2.095969\times FIGO.stage.4A\\ \; & -0.3352361\times FIGO.stage.4B-0.4496521\times ascites+2.317865\times PCI10 \end{array}$$

where:

age10 = age at diagnosis divided by 10;
  FIGO.Stage.4A = 1 if FIGO stage = 4A, FIGO.Stage.4A = 0 in other cases;
  FIGO.Stage.4B = 1 if FIGO stage = 4B, FIGO.Stage.4B = 0 in other cases;
  Ascites = 1 if there are ascites at diagnosis, else Ascites = 0;
  and PCI10 = Sugarbaker Peritoneal carcinosis index at diagnosis, divided by 10.

The results of this analysis showed that the two groups (PDS and early IDS surgery (<6 NACT cycles)) are not comparable with no significant overlap. The two groups show a significant difference in the PCI scores, with the early IDS score twice as high as the PDS group (22.7 versus 10.6, respectively), *p* <0.001. (Table 3 and Table S1). Furthermore, the five groups (PDS, surgery after three NACT cycles, after six cycles, after more than six cycles, and no surgery) are different; with essentially an initial tumor burden (PCI at diagnosis) and a different response to treatment (PCI evaluated in three or six courses with laparoscopy), data not shown.

A propensity score matching was used to reduce the bias when comparing the different NACT subgroups. The variables included in the matching included CA125 level (after logarithmic transformation), FIGO stage, age, ascites, and PCI score at diagnosis. The propensity score matching showed a difference between the three groups with some overlap.

**Figure 3:**
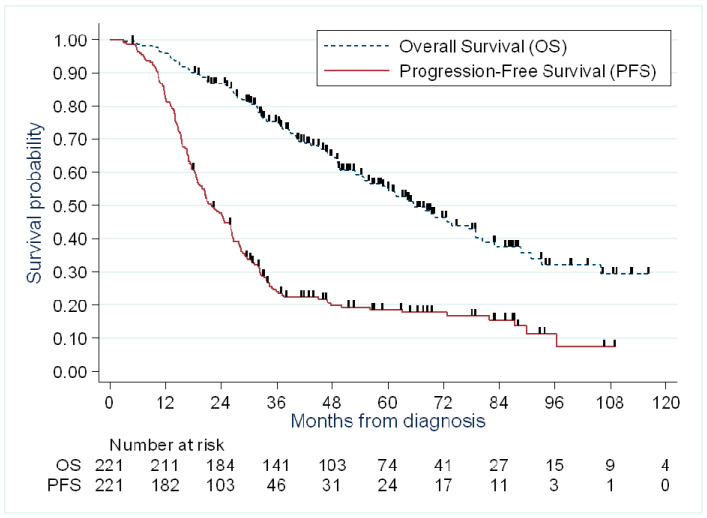
Survival outcomes in the entire cohort.

The Median follow-up period was 66.5 months (95% CI, 61.1–69.6 months). As shown in Table 4 and Figure 3, 179 patients presented disease progression with a median PFS of 21.9 months (95% CI, 18.9–26.1 months), and 107 patients died due to disease progression, leading to a median OS of 65.5 months (95% CI, 55.0–78.9% months).

Survival analysis of the PDS and NACT groups performed for the entire cohort without stratification on propensity score concluded to a median PFS of 29.2 months (95% CI, 26.0–34.3 months) and 18.9 months (95% CI, 16.7–21.9 months) in the PDS and NACT groups, respectively (HR = 1.99 (IC95%: 1.45–2.74))(*p* < 0.001) (Figure S1A). Median OS was 105.9 months (95%CI, 72.6 months—not reached) and 52.8 months (95%CI, 43.4–61.4 months) in the PDS and NACT groups, respectively (HR = 2.55 (1.62–4.02) *p* < 0.001) (Figure S2B).

The progression-free survival was significantly poorer in the early IDS surgery group (<6 NACT cycles) compared to PDS (HR = 1.55, 95%CI: 1.04–2.31, *p* = 0.03). However, this association was no longer significant after stratification on the quintile of the propensity score (HR = 0.86, 95%IC: 0.47–1.56, *p* = 0.61). Overall survival was not significantly associated with the treatment groups when comparing the early IDS group (<6 NACT cycles) and the PDS group, without or with stratification on the propensity score (HR = 1.63, 95%CI: 0.91–2.90, *p* = 0.10 and HR = 1.22, 95%CI: 0.54–2.78, *p* = 0.63) (Figure S1A,B).

Survival analysis of the NACT subgroups after propensity score matching found a significant decrease in the OS when delaying surgery. Compared with surgery after six cycles, early surgery (<six cycles) and delayed surgery (>six cycles) were associated with an HR of 0.81 (95%CI, 0.44–1.48) and 2.01 (95%CI, 1.07–3.75), respectively. The overall comparison of the three subgroups was statistically significant (*p* = 0.02).

## 4. Discussion

Our study compared early and long-term outcomes of advanced stage EOC patients treated via either PDS or NACT over ten years after a propensity score matching to answer whether the patients undergoing PDS are comparable to those undergoing NACT in a real-life setting. The comparison of the different groups—PDS versus NACT and the NACT groups among themselves—shows that the main, if not the only, factor that differentiates them is the initial tumor burden. The ovarian tumor burden is estimated by the Fagotti or Sugarbarker’s score (PCI). In our study, PCI is the only characteristic difference between PDS and NACT < six courses groups (Table 3).

The optimal timing for cytoreduction remains questionable in the literature. Based on extensive retrospective data (level 2–3 evidence), PDS was long considered the standard of care for advanced EOC treatment; however, there is no RCT to support this theory [30]. CC might require aggressive procedures that are sometimes associated with increased morbidity and delay or omission of adjuvant chemotherapy. Retrospective studies, Cochrane reviews, and four RCTs compared NACT and PDS.

RCTs concluded the no-inferiority of NACT with no difference in terms of survival (DFS and OS) and decreased surgical morbidity, and a trend towards a better quality of life [18,19,20,31,32,33,34,35,36].

Most of the trials randomized only patients eligible for both approaches, excluding patients with higher tumor burden inaccessible for PDS, thus not reflecting the whole range of disease severity at diagnosis. Furthermore, the comparability of the treatment groups (PDS versus NACT or IDS) is questionable in all the observational studies [37,38]. Booth et al. showed in their article how RCTs and observational studies are complementary in the evolution of medical evidence. RCTs are characterized by a very good internal validity and a reduced bias due to randomization. Still, they can present some limitations related to its applicability to the whole real population since patients participating in RCTs are selected according to the inclusion criteria. Observational studies are therefore complementary clinical practice resources.

Our analysis showed a median OS of 105.9 in the PDS group higher than the OS described by Luyckx et al. (74 months). In the NACT group, the median OS in our study was 52.8 months, in line with the findings of Luyckx (54 months), and is one of the highest reported values [20,31,32,33,35,37,38,39,40]. Despite our low PDS rate (38%), our median OS and PFS were 65.5 months and 21.9 months, respectively, for the whole cohort, which is concordant with data in literature [40,41].

A Will Rogers phenomenon [42] might explain the lower PDS rate and the enhanced OS in both PDS and NACT groups [43] by assigning patients with intermediate PCI to NACT while they can be PDS candidates. However, the fact that our entire cohort’s survival analysis without stratification is similar to the data in literature refutes this hypothesis [40]. Most studies exclude patients who do not undergo cytoreduction due to disease progression despite NACT. This induces a substantial bias since these patients form a part of the advanced ovarian cancer population and are initially managed with an intention to treat, but their disease extent precludes cytoreduction. Including this subgroup in the survival analysis reflects the actual image of the advanced epithelial ovarian cancer population. Most of the studies excluded patients who did not undergo surgery from their analysis, thus masking a part of the actual population (Table 5) [31,32,33,35,37,38,39,40].

We attribute our study’s high OS and PFS outcomes to the paradigm that differentiates patients receiving NACT from PDS patients. This is associated with a patient-centered pathway concordant with the international recommendations and comprises a systematic diagnostic laparoscopy with biopsies, surgery with the intent to achieve CC0 (90.6% of cases), and a systematic stay in the intensive care unit after surgery. Our propensity score stratification approach used to estimate the HR of NACT versus PDS might have partially corrected this bias. However, it also required us to exclude the highest quintile of the propensity score distribution, which only included patients with severe diseases treated with NACT. Furthermore, the propensity score may not wholly capture the indication bias, but it helps in reducing it. There is still no consensus on the ideal management strategy when both appear feasible, as Mueller et al. [40] emphasized. We believe that the differentiation between PDS patients and NACT patients should be based on initial tumor load reflected by the PCI score, CA 125 level, and the disease stage at diagnosis. This was highlighted by the significant difference in the PCI between the PDS and the early IDS surgery group (<6 NACT cycles) after the propensity score matching. This shows that patients in the “grey area zone” that cannot undergo PDS are different from and not comparable to patients eligible for PDS. This was also confirmed by the post hoc analysis subset analysis of the EORTC trial. It showed that patients with stage IIIC and less extensive metastatic tumors <4.5cm had better survival with PDS. Patients with stage IV disease and larger metastatic tumors >4.5cm had better survival with NACT [30].

In our study, only 9.4% (*n* = 20) and 28.6% (*n* = 61) of patients experienced intraoperative and postoperative complications, respectively, most of which were minor (grade 1–2), which are rarely reported in the literature. This observed morbidity rate seems acceptable, given the high rate of complex procedures reflected by the high Aletti’s scores. The risk of morbidity did not differ significantly between PDS and IDS, in contrast to the study by Mueller and the previously cited trials, which observed a higher incidence of severe postoperative morbidity after PDS [40].

The strength of our study resides in the extended follow-up with the long-term survival analysis for a large cohort of unselected patients with confirmed advanced stage HGSOC, including patients who never underwent surgery despite NACT (known to have poorer outcomes) [39,44]. Our cohort was also homogenous for the histologic subtype highlighting the most frequent and aggressive EOC subtype and homogenous for the therapeutic plans during the ten-year study period. Most importantly, the propensity score matching analysis helped limit selection bias and reflect the fundamental differences between populations. Our main limitation is the retrospective aspect of our study.

Evaluating initial tumor load during diagnostic laparoscopy appears to be the most crucial factor before deciding for PDS or NACT. Due to the considerable difference found between PDS and NACT patients, we think they represent two different populations requiring two different strategies based on the disease burden at diagnosis and the patient’s general status. Results of analysis comparing PDS and NACT are insufficient to reflect the real-life advanced EOC population. A detailed description of the patient’s characteristics at diagnosis and the outcome analysis of the whole population is required.

Advanced EOC is a heterogeneous population including PDS patients, NACT-IDS patients, and patients never making it to cytoreduction. NACT is best indicated for patients with high tumor load, in whom CC is deemed impossible, or in patients presenting severe comorbidities precluding PDS. A review of the different decision-making algorithms for advanced EOC management concludes that laparoscopic assessment is the most informative assessment tool [45].

Based on the literature and our findings, we think that the PDS rate should not be a quality indicator in advanced EOC management. Instead, survival data of the entire treated population, patients’ quality of life, morbidity, and rate of complications should be included as quality indicators, all of which put the patient back at the center of the management instead of the disease itself.

Putting the patient back at the center of the management is especially interesting in the cases where patients are eligible for both PDS and NACT-IDS with similar survival outcomes. In these cases, the patient’s general status, treatment-related morbidity, and expectations should be considered, and shared medical decisions could be attempted. Patient-centered priorities assessment tools are currently being evaluated to help shared medical decisions in ovarian cancer.

## 5. Conclusions

Advanced HGSOC is a heterogeneous population in which trials proved the non-inferiority of NACT and IDS compared to PDS in patients eligible for both. However, patients inoperable straightaway should be differentiated from candidates to PDS based on many factors, out of which the tumor burden is a major one. Putting the patient back at the center of the treatment plan by focusing on survival data, the patient’s quality of life, and post-treatment morbidity seems great value than the rate of PDS as quality indicators for advanced EOC management.

## Figures and Tables

**Table 1:** Patients and disease characteristics.

Characteristics	All Included Patients *N* = 221 Patients	PDS Group *N* = 84 Patients	NACT Group *N* = 137 Patients	*p*-Value
Age (years)				
Median (range)	63.2 (22.9–88.0)	64.3 (31.2–84.5)	62.6 (22.9–88.0)	
Mean (SD)	62.3 (10.6)	63.5 (10.2)	61.6 (10.8)	0.22
Body Mass Index (kg/m^2^)				
Median (range)	24.8 (15.4–59.1)	24.7 (16.4–59.1)	24.9 (15.4–44.1)	
Mean (SD)	26.0 (5.8)	25.8 (6.0)	26.1 (5.7)	0.76
CA 125 (UI/mL)				
Median (range)	913 (4–29376)	287 (4–11870)	1160 (15–29376)	0.002
Mean (sd)	1815 (3072)	1077 (1811)	2249 (3549)	0.001
FIGO stage				
IIIC, *n* (%)	181 (81.9)	79 (94.0)	102 (74.5)	
IVA, *n* (%)	21 (9.5)	2 (2.4)	19. (13.9)	
IVB, *n* (%)	19 (8.6)	3 (3.6)	16 (11.6)	
Ascites, *n* (%)	83 (37.6)	27 (32.1)	56 (40.9)	0.19
Peritoneal Carcinomatosis Index (PCI)				
Median (range)	20 (0–39)	10 (1–24)	25 (0–39)	
Mean (SD)	18.7 (10.5)	10.6 (6.7)	24.4 (8.8)	0.0001
≤10, *n* (%)	53 (24)	45 (53.6)	8 (5.8)	
11–24, *n* (%)	91 (41.2)	39 (46.4)	52 (38.0)	
≥25, *n* (%)	77 (34.8)	0 (0)	77 (56.2)	

PDS: Primary Debulking Surgery; IDS: Interval Debulking Surgery; NACT: Neoadjuvant Chemotherapy; FIGO: 2018 International Federation of Gynecology and Obstetrics; SD: Standard Deviation.

**Table 2:** Surgical characteristics, intraoperative and postoperative morbidity.

Characteristics	All Included Patients *N* = 221 Patients	PDS Group *N* = 84	NACT Group *N* = 129	*p*-Value
Peritoneal Carcinomatosis Index at surgery				0.34
Median (range)	10 (0–25)	10 (1–24)	10 (0–25)	
Mean (SD)	11.2 (7.1)	10.6 (6.7)	11.6 (7.3)	0.34
Type of surgical act (1)				0.01
Standard, *n* (%)	67 (31.5)	26 (31.0)	41 (31.8)	
Radical, *n* (%)	36 (16.9)	22 (26.2)	14 (10.9)	
Supra-radical, *n* (%)	110 (51.6)	36 (42.9)	74 (57.4)	
Para-aortic lymphadenectomy, *n* (%)	198 (93.0)	79 (94.0)	119 (92.2)	0.62
Bowel Resection, *n* (%)	92 (43.2)	47 (56.0)	45 (34.9)	0.002
Aletti’s complexity score (2)				0.82
Low, *n* (%)	5 (2.3)	1 (1.2)	4 (3.1)	
Intermediate, *n* (%)	117 (54.9)	47 (56.0)	70 (54.3)	
High, *n* (%)	91 (42.7)	36 (42.9)	55 (42.6)	
Resection quality				0.001
CC0, *n* (%)	193 (90.6)	82 (97.6)	111 (80.0)	
CC1, *n* (%)	12 (5.6)	0 (0)	12 (9.3)	
CC2, *n* (%)	7 (3.3)	1 (1.2)	6 (4.7)	
CC3, *n* (%)	1 (0.5)	1 (1.2)	0 (0)	
Duration of surgery (min)				
Median (range)	300 (125–665)	300 (125–665)	300 (150–590)	
Mean (sD)	318.3 (96.0)	324.4 (100.6)	314.3 (93.1)	0.48
Estimated blood loss (mL)				
Median (range)	1000 (0–7000)	950 (0–6000)	1000 (100–7000)	
Mean (SD)	1281 (1126)	1241.5 (1130)	1311 (1131)	0.75
Transfusion, *n* (%)	45 (21.1)	13 (15.5)	32 (24.8)	0.10
Duration of hospital stay				
Median (range)	10 (3–50)	11 (3–32)	10 (4–50)	
Mean (SD)	11.6 (6.0)	11.9 (5.5)	11.4 (6.3)	0.58
Intra-operative morbidity, *n* (%)	20 (9.4)	8 (9.5)	12 (9.3)	0.96
Postoperative morbidity, *n* (%)	61 (28.6)	27 (32.1)	34 (26.4)	0.36
Morbidity (intra or postoperative) Clavien-Dindo grade				0.29
1–2, *n* (%)	33 (15.5)	13 (15.5)	20 (15.5)	
3A, *n* (%)	14 (6.6)	9 (10.7)	5 (3.9)	
3B, *n* (%)	13 (6.1)	5 (6.0)	8 (6.2)	
4, *n* (%)	1 (0.5)	0 (0)	1 (0.8)	

(1) Type of surgical act was classified in three categories: -Standard, including hysterectomy, bilateral adnexectomy with total omentectomy including supracolic-omentum, appendicectomy, para-aortic, and pelvic lymphadenectomy with or without peritonectomy; -Radical: standard surgery with additional recto-sigmoidectomy; -Supra-radical surgery, a standard or radical surgery, with additional extensive peritonectomy including partial diaphragm’s resection, or resection of subcapsular liver metastases, cholecystectomy, splenectomy, or another bowel resection (frequently described as upper abdominal surgery). (2) Aletti’s complexity score was classified into three categories considering the following cut-offs: low ≤ 3, intermediate 4 to 7, high ≥ 8.

**Table 3:** Patients’ characteristics of the PDS and the early IDS (NACT < 6 cycles) groups.

Characteristics	PDS Group *N* = 84 Patients	NACT Group: <6 Courses *N* = 50 Patients	*p*-Value
Age (years)			
Median (range)	64.3 (31.2–84.5)	62.9 (22.9–88)	
Mean (SD)	63.5 (10.2)	62.1 (12.1)	0.49
Body Mass Index (kg/m^2^)			
Median (range)	24.7 (16.4–59.1)	24.4 (16.9–40)	
Mean (SD)	25.8 (6.0)	25.6 (5.5)	0.80
CA 125 (UI/mL)			
Median (range)	287 (4–11,870)	936 (14.5–5568)	
Mean (sd)	1077 (1811)	1482.4 (1470)	0.19
FIGO stage			
IIIC, *n* (%)	79 (94%)	45 (90%)	
IVA, *n* (%)	2 (2%)	3 (6%)	
IVB, *n* (%)	3 (4%)	2 (4%)	0.59
Ascites, *n* (%)	27 (32%)	20 (40%)	0.36
Peritoneal Carcinomatosis Index (PCI)			
Median (range)	10 (1–24)	24 (3–39)	
Mean (SD)	10.6 (6.7)	22.7 (7.8)	<0.001
≤10, *n* (%)	45 (54%)	3 (6%)	
11–24, *n* (%)	39 (46%)	21 (42%)	
≥25, *n* (%)	0 (0%)	23 (46%)	

**Table 4:** Overall and progression-free survival of the PDS population, NACT population, and all NACT subgroups.

Outcomes	All Included Patients	Treatment Strategy		Timing of Surgery in the NACT Group		
		PDS	NACT	<6 Courses	6 Courses	>6 Courses
	*N* = 221	*N* = 84	*N* = 137	*N* = 50	*N* = 50	*N* = 29
Progression-free survival						
Number of events	179	56	123	43	45	27
Median PFS (95%CI)	21.9 (19–26)	29.2 (26.2–34.3)	18.9 (16.7–21.9)	22.1 (17.7–28.3)	18.1 (14.6–24.3)	16.6 (10.7–21.2)
12 month PFS (95%CI)	83% (77–87)	94% (86–98)	76% (68–82)	82% (68–90)	82% (68–90)	59% (39–74)
24 month PFS (95%CI)	48% (41–54)	63% (51–72)	38% (30–47)	48% (34–61)	38% (25–51)	26% (12–43)
36 month PFS (95%CI)	24% (19–30)	39% (28–49)	15% (10–22)	23% (12–35)	14% (6–25)	4% (0–18)
Overall survival						
Number of deaths	107	25	82	22	30	23
Median OS (95%CI)	65.5 (55–79)	105.9 (72.6–NR)	52.8 (43.4–61.4)	69.2 (48.8–NR)	53.1 (38.9–77.4)	40.3 (24.8–54.9)
12 month OS (95%CI)	96% (92–98)	98% (91–99)	95% (90–98)	98% (87–100)	94% (83–98)	93% (75–98)
24 month OS (95%CI)	87% (82–91)	93% (85–97)	83% (76–89)	92% (80–97)	86% (73–93)	72% (52–85)
36 month OS (95%CI)	75% (68–80)	89% (80–94)	66% (58–74)	77% (62–86)	67% (52–78)	53% (32–69)

PDS: Primary Debulking Surgery; NACT: Neoadjuvant Chemotherapy; IDS: Interval Debulking Surgery; 95%CI: 95% Confidence Interval; NR: Not Reached.

**Table 5:** Literature Data.

Study	Design	Timing of Surgery	N (%PDS)	Median OS, by Subgroup (Months)	Median OS, Entire Population (Months)	Median Follow-Up (Months)
Vergote et al., 2010 [32]	Randomized FIGO IIIC to IV comparing PDS to IDS	PDS IDS	336 334	29 30	DNS	34
Kehoe et al., 2015 [31]	Randomized FIGO II to IV comparing PDS to IDS	PDS IDS	276 274	22.6 24.1	DNS	52.8
Fagotti, 2020 [33]	Randomized FIGO IIIC and IV PDS vs. IDS	PDS IDS	84 74	41 43	DNS	59
Chi et al., 2012 [35]	Retrospective, monocenter FIGO IIIC to IV	PDS IDS	285 (90%) 31	50 37	DNS	DNS
Luyckx et al., 2012 * [37]	Retrospective multicenter, FIGO IIIC to IV	PDS IDS	190 (36%) 337	74 54	NR	49
Mueller et al., 2016 [40]	Retrospective, monocenter FIGO III to IV,	PDS IDS	432 (74%) 154	71.7 42.9	63.2	44.4
Rauh-Hain et al., 2017 [38]	Retrospective, multicenter FIGO IIIC to IV	PDS IDS	19,836 (86%) 3126	37.3 32.1	DNS	56.5
Kessous et al., 2017 [39]	Retrospective, monocenter FIGO III to IV	PDS Including PDS+ CC0 IDS	136 (52%) 55 127	60.2 106 48.8	DNS	DNS
Present Series	Retrospective, monocenter FIGO IIIC to IV	PDS NACT	84 (38%) 137	105.9 52.8	65.5	66.5

OS: Overall Survival; PDS: Primary Debulking Surgery; IDS: Interval Debulking Surgery; FIGO: Federation International of Gynecology and Obstetrics. DNS: Data not shown; NR: Not Reached * A few patients of the current study recruited patients from January 2007 to December 2017 at Oscar Lambert Center were included in the multicenter study published by Luyckx et al., reporting patients recruited between January 2003 and December 2007.

## Data Availability

The datasets used and analyzed during the current study are available from the corresponding author on reasonable request.

## Supplementary Materials

The following are available online at https://www.mdpi.com/article/10.3390/cancers13194925/s1, Figure S1: Survival Analysis in PDS and early IDS surgery (<6 NACT cycles) groups. A: Progression-free survival. B: Overall survival, Table S1: Surgical characteristics, intraoperative and postoperative morbidity of the PDS and the NACT < 6 cycles.
