# Supplementary material for: A New Paradigm in Managing Advanced Ovarian Cancer: Differentiating Patients Requiring Neoadjuvant Treatment from Primary Cytoreduction
Source: Cancers (Basel). 2021 Sep 30;13(19):4925. doi: 10.3390/cancers13194925 (PMC8508489; doi:10.3390/cancers13194925)
Supplement: Supplementary file 1 [file cancers-13-04925-s001.zip › cancers-1356277-supplementary.pdf]

Supplementary: Analysis comparing the PDS and the Early IDS group (NACT<6 cycles)

Table S1. Surgical characteristics, intraoperative and postoperative morbidity of the PDS and the NACT<6 cycles.

Characteristics	PDS Group N= 84 patients	NACT Group: <6 courses N = 50 patients	P-value
**Age (years)**			
**Median (range)**	64.3 (31.2 - 84.5)	62.9 (22.9 - 88)	
**Mean (SD)**	63.5 (10.2)	62.1 (12.1)	0.49
**Body Mass Index (kg/m²)**			
**Median (range)**	24.7 (16.4 - 59.1)	24.4 (16.9 - 40)	
**Mean (SD)**	25.8 (6.0)	25.6 (5.5)	0.80
**CA 125 (UI/mL)**			
**Median (range)**	287 (4 - 11870)	936 (14.5 - 5568)	
**Mean (sd)**	1077 (1811)	1482.4 (1470)	0.19
**FIGO stage**			
**IIIC, n (%)**	79 (94%)	45 (90%)	
**IVA, n (%)**	2 (2%)	3 (6%)	
**IVB, n (%)**	3 (4%)	2 (4%)	0.59
**Ascites, n (%)**	27 (32%)	20 (40%)	0.36
**Peritoneal Carcinomatosis Index (PCI)**			
**Median (range)**	10 (1 - 24)	24 (3 - 39)	
**Mean (SD)**	10.6 (6.7)	22.7 (7.8)	<0.001
**≤10, n (%)**	45 (54%)	3 (6%)	
**11-24, n (%)**	39 (46%)	21 (42%)	
**≥25, n (%)**	0 (0%)	23 (46%)	

Characteristics	PDS Group N= 84	<6 courses of NACT Group N = 50	P-value
**Peritoneal Carcinomatosis Index at surgery**			0.51
Median (range)	10 (1 - 24)	10 (0 - 25)	
Mean (SD)	10.6 (6.7)	11.4 (6)	

Characteristics	PDS Group N= 84	<6 courses of NACT Group N = 50	P-value
**Type of surgical act** (1)			0.15
Standard, n (%)	26 (31%)	12 (24%)	
Radical, n (%)	22 (26%)	8 (16%)	
Supra-radical, n (%)	36 (43%)	30 (60%)	
**Para-aortic lymphadenectomy**, n (%)	79 (94%)	46 (92%)	0.73
**Bowel Resection,** n (%)	47 (56%)	16 (32%)	0.007
**Aletti's complexity score** (2)			0.83
Low, n (%)	1 (1%)	0 (0%)	
Intermediate, n (%)	47 (56%)	30 (60%)	
High, n (%)	36 (43%)	20 (40%)	
**Resection quality**			0.12
CC0, n (%)	82 (98%)	46 (92%)	
CC1, n (%)	0 (0%)	1 (2%)	
CC2, n (%)	1 (1%)	3 (6%)	
CC3, n (%)	1 (1%)	0 (0%)	
**Duration of surgery (min)**			0.25
Median (range)	300 (125 - 665)	295 (165 - 590)	
Mean (SD)	324.4 (100.6)	303.4 (89.2)	
**Estimated blood loss (mL)**			0.30
Median (range)	950 (0 - 6000)	1000 (100 - 2800)	
Mean (SD)	1241.5 (1130)	1022 (651.1)	
**Transfusion,** n (%)	13 (16%)	10 (20%)	0.50
**Duration of hospital stay**			0.11
Median (range)	11 (3 - 32)	10 (5 - 30)	
Mean (SD)	11.9 (5.5)	10.4 (4.6)	
**Intra-operative morbidity,** n (%)	8 (10%)	7 (14%)	0.43
**Postoperative morbidity,** n (%)	27 (32%)	14 (28%)	0.62

Characteristics	PDS Group N= 84	<6 courses of NACT Group N = 50	P-value
**Morbidity (intra or postoperative) Clavien-Dindo grade**		NR=36	1.00
1-2, n (%)	13 (16%)	7 (14%)	
3A, n (%)	9 (11%)	4 (8%)	
3B, n (%)	5 (6%)	3 (6%)	
4, n (%)	0 (0%)	0 (0%)	

Figure S1. Survival Analysis in PDS and early IDS surgery (<6 NACT cycles) groups. A: Progression-free survival. B: Overall survival.

Figure S-1A : HR=1.63, 95%CI: 0.91-2.90, p=0.10

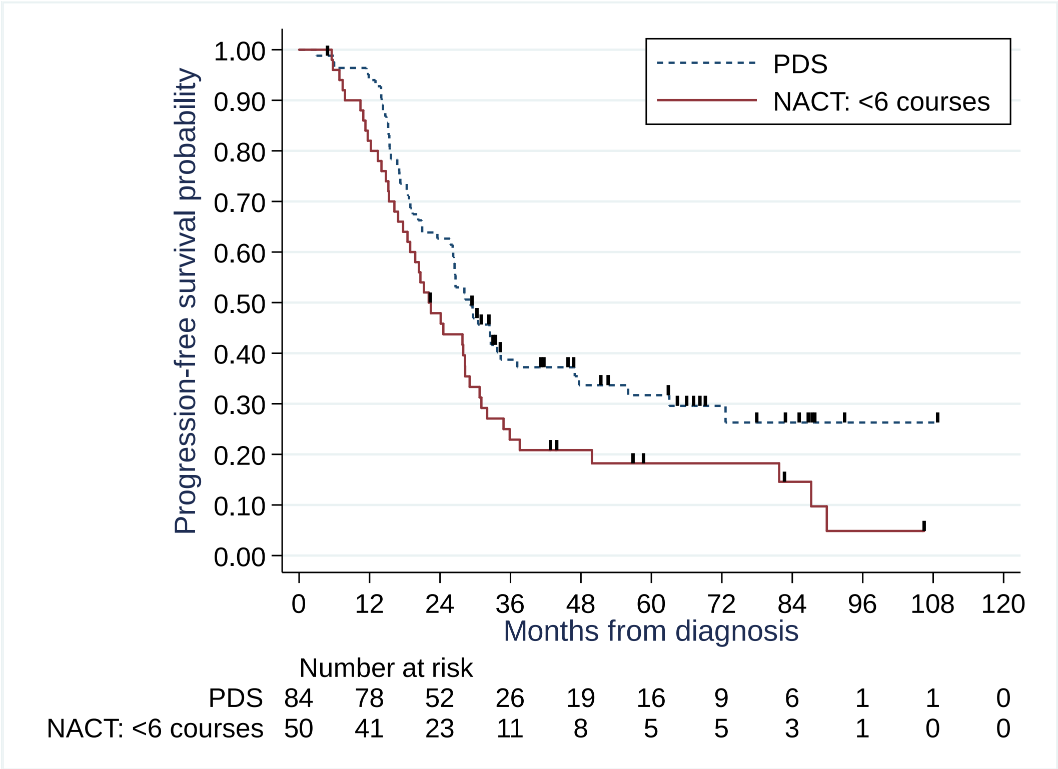

Figure S-1B: HR=1.22, 95%CI: 0.54-2.78, p=0.63.

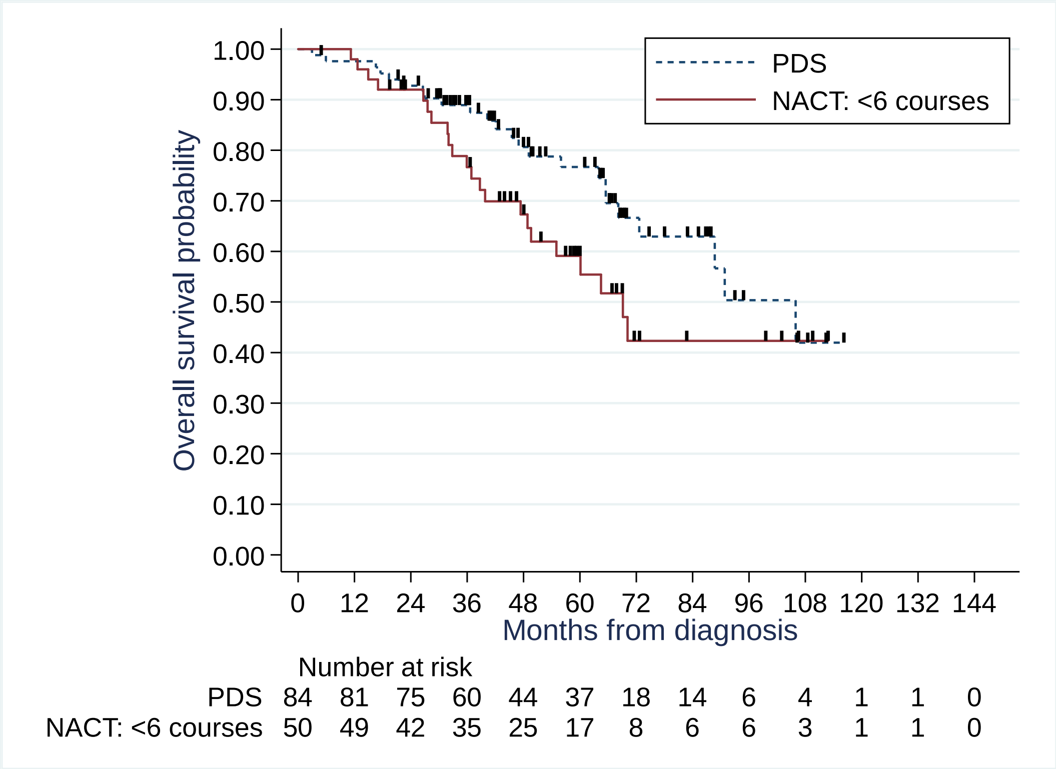

Figure S-1: Survival Analysis in PDS and NACT groups. A: Progression-free survival. B: Overall survival.

Figure S-1A : HR=1.99 [IC95%: 1.45-2.74])(p<0.001)

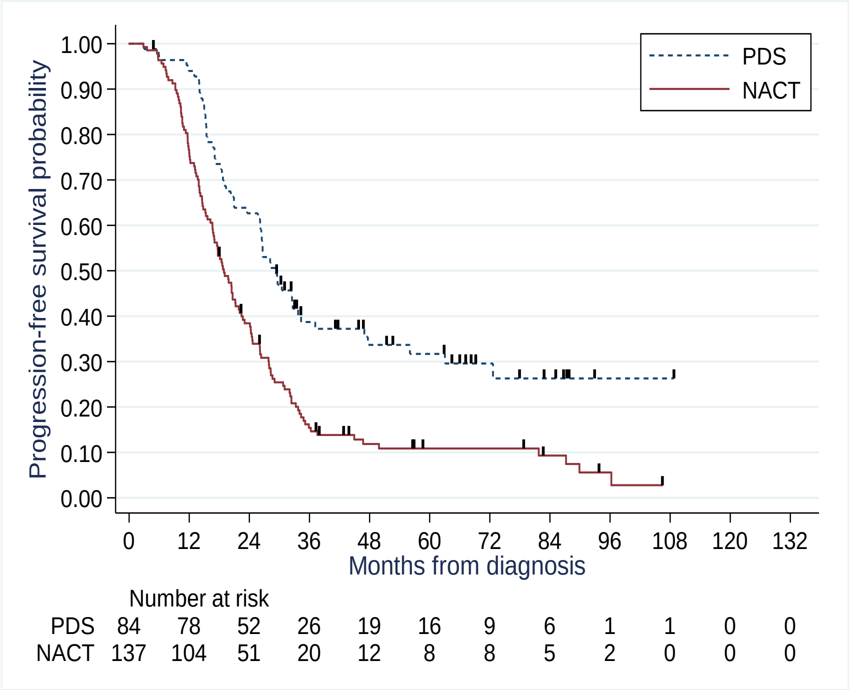

Figure S-1B: HR=2.55 [1.62-4.02] p<0.001

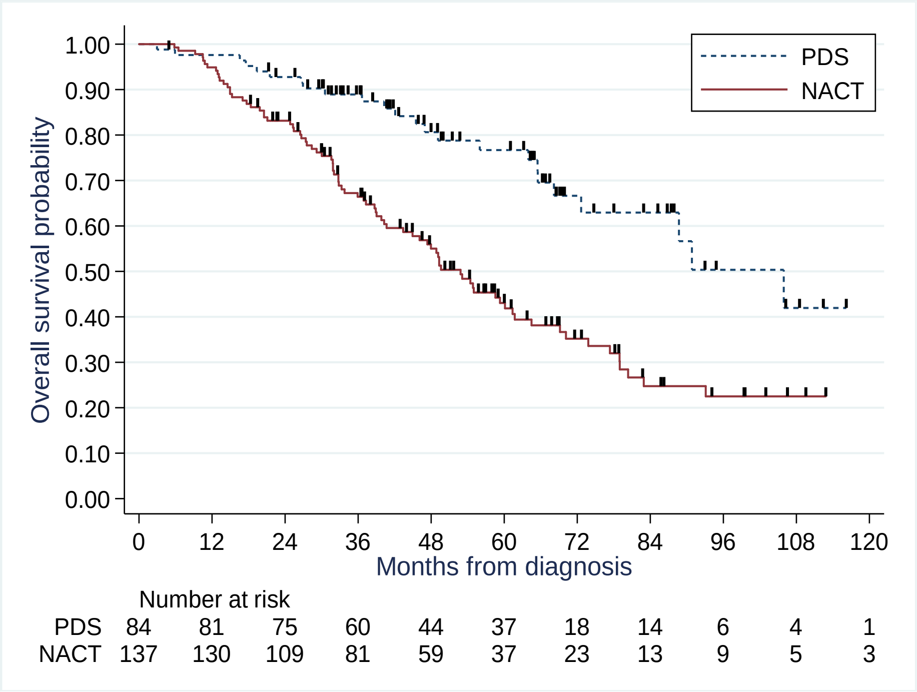
PDS
NACT
Overall survival probability
1.00
0.90
0.80
0.70
0.60
0.50
0.40
0.30
0.20
0.10
0.00
0
12
24
36
48
60
72
84
96
108
120
Months from diagnosis
Number at risk
PDS 84 81 75 60 44 37 18 14 6 4 1
NACT 137 130 109 81 59 37 23 13 9 5 3
